# Dose Comparison of Flowable Gelatin Hemostatic Matrix for Bleeding Loss in Primary Total Knee Arthroplasty

**DOI:** 10.7759/cureus.64780

**Published:** 2024-07-17

**Authors:** Shogo Matsuda, Masashi Miyazaki, Masashi Hirakawa, Yu Nagashima, Hiroya Akase, Nobuhiro Kaku

**Affiliations:** 1 Orthopedic Surgery, Oita University, Yufu, JPN; 2 Orthopedics, Oita University, Yufu, JPN

**Keywords:** hemoglobin, total blood loss, hemostatic agent, bleeding control, total knee arthroplasty

## Abstract

Introduction

Intraoperative and postoperative bleeding in total knee arthroplasty (TKA) affects postoperative outcomes. Although the hemostatic effect of a flowable gelatin hemostatic matrix (FGHM) is known across several surgical fields, its effectiveness on TKA remains controversial. This study aimed to compare the amount of bleeding across three groups treated with different doses of FGHM in TKA.

Methods

Overall, 122 knee joints of patients who underwent unilateral primary TKA were included and divided into three groups according to FGHM dose: absence of FGHM (control group, N=48), administration of 5 mL of FGHM (5 mL group, N=46), and administration of 8 mL of FGHM (8 mL group, N=38). Total hemoglobin (Hb) loss, drain output, hidden blood loss (HBL), calculated total blood loss (TBL) on the first postoperative day (POD1) and one week postoperatively (POD7), postoperative flexion angle at one week and discharge, and incidence of postoperative deep venous thrombosis (DVT) were assessed.

Results

At POD1, the mean total Hb losses were 6.3±3.1g (control group), 5.5±3.3g (5 mL group), and 5.3±2.5g (8 mL group), with no significant differences. At POD7, the mean Hb losses were 9.1±4.6g (control), 8.7±3.6g (5 mL), and 8.3±4.0g (8 mL), also with no significant differences. Mean drain outputs and HBLs showed no significant differences among groups. While there was a decreasing trend in TBL with higher FGHM doses, it was not statistically significant at either POD1 or POD7. There were no statistically significant differences in the mean postoperative flexion angle at POD7 or discharge among the groups (99.7±12.6°, 95.7±12.5°, 98.3±13.8° at POD7; 115.9±9.7°, 113.8±9.6°, 116.6±9.2° at discharge). Of these, only one patient in the 8 mL group developed proximal DVT.

Conclusion

Despite a trend towards decreased bleeding with FGHM, no significant differences were found among the three groups. However, the clinical utility of this hemostatic agent for reducing blood loss after primary TKA remains still unclear.

## Introduction

Total knee arthroplasty (TKA) is a useful treatment for diseases, such as osteoarthritis and rheumatoid arthritis. However, perioperative complications can lead to prolonged hospitalization and decreased patient satisfaction. Postoperative hemorrhage is a complication that may cause worsening of postoperative pain, local swelling, and decreased postoperative range of motion [1]. There are various methods to reduce blood loss, such as intraoperative electrocoagulation, tranexamic acid administration, and minimally invasive surgery. However, more effective hemostatic methods necessitate further consideration.

A flowable gelatin hemostatic matrix (FGHM), which is a combination of a bovine-derived gelatin matrix, serves as an adhesive, sealant, and human-derived thrombin. Its mixture acts as a hemostatic and sealing agent, decreasing surgical field bleeding [2]. Its usefulness has been reported in cardiovascular surgery, neurology, and spine surgery [2-5]. While some studies have reported decreased postoperative blood loss upon using FGHM in TKA, others have reported no change [6-12]. Additionally, few studies have compared different FGHM doses.

The purpose of this study was the following threefold: 1. Evaluate the efficacy of different doses of FGHM in reducing intraoperative and postoperative bleeding in patients undergoing primary TKA; 2. Compare the amount of total hemoglobin loss, drain output, hidden blood loss, and calculated total blood loss among three groups: no FGHM (control group), 5 mL FGHM group, and 8 mL FGHM group; 3. Assess the impact of FGHM administration on postoperative knee flexion angle and the incidence of postoperative complications, specifically deep venous thrombosis (DVT). We hypothesized that higher doses of FGHM would be more effective than no dose or lower doses in reducing blood loss in TKA and would affect the postoperative knee flexion angle.

## Materials and methods

Study design and patients

This study provides a historical perspective on non-invasive monitoring. The study protocol was approved by the Institutional Review Board of Oita University Hospital (IRB registration number: 11000787). Informed consent was obtained in the form of an opt-out. The need for informed consent from individual patients was waived by the ethics committee of our institution.

This study included 132 knee joints of patients who underwent unilateral primary TKA at our hospital between July 2013 and October 2022. Patients were stratified into three groups: absence of FGHM (control group, N=48), administration of 5 mL of FGHM (Floseal🄬; Baxter Healthcare Corporation, Deerfield, IL, USA) (5 mL group, N=46), and administration of 8 mL of FGHM (Surgiflo🄬; Ethicon Incorporated, Raritan, NJ, USA) (8 mL group, N=38). The assignment to the treatment arm was predicted according to the predetermined study duration. The control, 5 mL, and 8 mL groups were enrolled between July 2013 and March 2017, between November 2016 and January 2021, and between June 2021 and October 2022, respectively. The following diseases that may affect the amount of bleeding as patient predisposition were considered as exclusions: patients who were currently prescribed antiplatelet or anticoagulant medications; those with coagulopathy (platelet counts below 100,000 or abnormal prothrombin time (PT), activated partial thrombin time, and international normalized ratio of PT exceeding 1.4); individuals with a medical history of liver disease; and patients who underwent lateral release surgery.

Surgical techniques of primary TKA

In all cases, TKA implants were cemented with TKA, which was either a Future Knee (Posterior-Stabilized Prosthesis; Teijin-Nakashima, Okayama, Japan) or Persona (Posterior-Stabilized Prosthesis; Zimmer-Biomet, Warsaw, IN, USA). All procedures were conducted under general anesthesia. A tourniquet was applied prior to skin incision at 250 mmHg and released upon completing skin closure. Intra-and extramedullary alignment rods were utilized for femoral and tibial resection, respectively. All patients underwent patellar resurfacing. Following implant cementation, FGHM was administered to the soft tissues encompassing the posterior articular capsule and exposed bone marrow before insertion. Following FGHM application, pressure was applied to the area with moistened gauze for 2 minutes, and any residual FGHM was rinsed away with a saline solution. After the articular cuspule was closed, intra-articular administration of 3 g tranexamic acid (TXA) was performed via the drain tube, which was subsequently clamped for two to three hours postoperatively. After clamp release, full pressure was applied, and the drain was removed first postoperative day. All patients were administered subcutaneous injections of 2000 IU enoxaparin for 10 days to prevent DVT, commencing on the second postoperative day. All patients underwent lower extremity echography on postoperative day 7 to evaluate DVT.

Outcome evaluation

The primary outcomes of this study included total hemoglobin (Hb) loss, drain output, hidden blood loss (HBL), calculated total blood loss (TBL) on the first postoperative day (POD1) and one week postoperatively (POD7), flexion angle at one week postoperatively and during discharge, and the incidence of postoperative DVTs.

Calculation of blood loss

Preoperative circulating blood volume and TBL were calculated according to the method [13]. The formula can be summarized as preoperative circulating blood volume=k1×height (m) + k2×weight (kg) + k3 (k is a constant, k1=0.3669, k2=0.03219, and k3=0.6041 for males and 0.3561, 0.03308, and 0.1833 for females, respectively), and TBL=1000×total blood volume×(total Hb loss)/preoperative Hb. Hb loss was calculated via the method of Good and Kalairajah [14,15], as follows: Total Hb loss(g)=preoperative circulating blood volume×(preoperative Hb×postoperative Hb)×0.001. HBL was calculated as follows: HBL=TBL(ml) - drain output(ml). 

Statistical analysis

The sample size was developed using an alpha of 0.05 and a beta of 0.80. Considering that 10% of the patients would be lost to follow-up and 10% would be excluded, the study aimed to enroll 80 patients.

Tukey-Kramer's test was utilized for statistical analysis of the distribution of demographic and preoperative and postoperative clinical data among the three groups. Differences in descriptive data, including sex and DVT incidence, between the three groups were compared using chi-square test. Differences were considered statistically significant at P<0.05. All statistical comparisons were done using SPSS version 25 (IBM Corp., Armonk, NY, USA). 

## Results

Comparison of clinical features

Table 1 demonstrates the demographic data, revealing that all variables were similar across groups, except for age and operative time.

**Table 1:** Details of the patients in the three groups

	Control Group (n=48)	5 ml Group (n=46)	8 ml Group (n=38)	P value
Age (yr)	70.8±10.3	75.9±8.5	74.6±7.2	0.016
Male:female	7:41	14:32	6:32	0.114
BMI (kg/m^2^)	26.8± 3.9	25.6±3.9	25.9±4.2	0.278
Operative time (min）	123.7±10.0	123.0±10.8	116.5±12.2	0.006
Preoperative-Hb (g/dL）	13.3±1.0	13.3±1.4	12.9±1.4	0.319
Preoperative circulating blood volume (L）	3.53±0.63	3.62±0.52	3.50±0.56	0.565
Preoperative flexion angle (deg）	118.1±15.7	121.7±13.0	122.1±14.5	0.355
Length of hospital stay (day）	24.9±3.3	25.7±5.0	26.7±4.3	0.185

Perioperative blood loss in patients

At POD1, the mean total Hb losses were 6.3±3.1 g, 5.5±3.3 g, and 5.3±2.5 g in the control, 5 mL, and 8 mL groups, respectively (Table 2). Despite a decreasing trend with increasing FGHM dose, the difference was not statistically significant. Similarly, the mean total Hb losses at POD7 were 9.1±4.6 g, 8.7±3.6 g, and 8.3±4.0 g in the control, 5 mL, and 8 mL groups, respectively, without statistically significant differences (Table 2).

**Table 2:** Postoperative total hemoglobin (Hb) loss POD1: first postoperative day, POD7: one week postoperative

	Control	5 ml	8 ml	P value
Total Hb loss, POD1 (g)	6.3±3.1	5.5±3.3	5.3±2.5	0.528
Total Hb loss, POD7 (g)	9.1± 4.6	8.7±3.6	8.3±4.0	0.543

The mean drain outputs were 204.3±131.8 mL, 224.2±183.0 mL, and 218.9±154.9 mL in the control, 5 mL, and 8 mL groups, respectively, without statistically significant differences. The mean HBLs at POD1 were 261.7±219.7 mL, 193.7±240.4 mL, and 185.3±231.5 mL in the control, 5 mL, and 8 mL groups, respectively. At POD7, the mean HBLs were 468.3±302.2 mL, 432.0±246.1 mL, and 420.9±265.9 mL in the control, 5 mL, and 8 mL groups, respectively. However, the differences were not statistically significant. Despite a decreasing trend in TBL with FGHM dose, the differences were not statistically significant at both POD1 and POD7 (466.0±204.4 mL, 417.9±245.3 mL, 404.5±185.6 mL; and 672.5±302.7 mL, 656.2±258.2 mL, 629.4±292.2 mL, respectively) (Table 3).

**Table 3:** Postoperative drainage volume, hidden blood loss, and total blood loss POD1: first postoperative day, POD7: one week postoperative

	Control	5 ml	8 ml	P value
Drain drainage volume (ml)	204.3±131.8	224.2±183.0	218.9±154.9	0.820
Hidden blood loss, POD1 (ml)	261.7± 219.7	193.7±240.4	185.3±231.5	0.230
Hidden blood loss, POD7 (ml)	468.3±302.2	432.0±246.1	420.9±265.9	0.696
Total blood loss, POD1 (ml)	466.0±204.4	417.9±245.3	404.5±185.6	0.366
Total blood loss, POD7 (ml)	672.5±302.7	656.2±258.2	629.4±292.2	0.783

Postoperative flexion angle

The mean postoperative flexion angle demonstrated no statistically significant difference at POD7 or discharge (99.7±12.6°, 95.7±12.5°, 98.3±13.8°, and 115.9±9.7°, 113.8±9.6°, 116.6±9.2°, respectively) (Table 4).

**Table 4:** Postoperative flexion angle POD7: one week postoperative

	Control	5 ml	8 ml	P value
Flexion angle at POD7 (degree)	99.7±12.6	95.7±12.5	98.3±13.8	0.314
Flexion angle at discharge (degree)	115.9± 9.7	113.8±9.6	116.6±9.2	0.543

Complication

Only one patient in the 8 mL group had proximal DVT on lower extremity echography at one week postoperatively. Subsequently, apixaban-induced thrombolytic therapy was administered, leading to thrombus dissolution. Notably, no mortality attributable to any cause was observed across the three groups.

## Discussion

This study demonstrated a trend toward less bleeding with FGHM; however, the difference in blood loss and postoperative range of motion was not statistically significant.

The effectiveness of FGHM during TKA remains controversial. Bae et al. revealed that using FGHM significantly decreased drain output (p=0.010) and significantly reduced the necessity of blood transfusion in TKA [9]. Velyvis et al. reported that, compared to the control group, 5 mL and 10 mL of FGHM significantly reduced the drain output in the 10 mL group (p<0.001) [6]. In a prospective randomized trial, Suarez et al. revealed that using FGHM led to decreased calculated blood loss (p=0.02) and drain output (p=0.008) during primary TKA. However, Kim et al. reported that FGHM did not decrease blood loss, as measured based on drain output. They concluded that using FGHM remains inconclusive [10]. In this study, there was no significant difference in blood loss among the three groups. One difference from previous literature was the administration of TXA in the drain, which may be one of the reasons for this difference.

According to previous studies, the intra-articular injection of TXA is safe and reduces bleeding [16-19]. Tammachote et al. compared the amount of bleeding in groups that received 3 g and 500 mg of TXA intra-articularly. It was concluded that the 3 g group had reduced bleeding compared to the 500 mg group, and there was no difference in thromboembolic complications [19]. In the present study, TXA and FGHM were used simultaneously for reducing perioperative blood loss. Despite no significant difference being found in perioperative blood loss, the hemostatic effect of TXA may have prevented the difference in blood loss among the three groups.

Previous studies have presented conflicting perspectives on the necessity of releasing an air tourniquet while administering an FGHM. Velyvis et al. reported that there was no discernible contrast in the timing of FGHM utilization since the postoperative drain volume and transfusion usage rates decreased before and after air tourniquet release during FGHM administration [6]. Bae et al. revealed a decrease in postoperative blood loss after applying FGHM under an air tourniquet [9,10]. Nevertheless, considering the mechanism of action of FGHM, it may be more efficacious to release an air tourniquet and apply it to curtail bleeding. FGHM is a composite of a bovine-derived gelatin matrix and human thrombin components. Upon its application to a hemorrhagic area, human thrombin catalyzes the conversion of fibrinogen to fibrin, thereby accelerating blood coagulation. Moreover, the gelatin matrix expands when exposed to moisture, generating a tamponade effect that aids hemorrhage arrest [2]. One plausible explanation for the absence of variance in the degree of bleeding between the FGHM-treated and non-treated cohorts in our study is that FGHM administration without releasing the air tourniquet may not have been sufficient to suppress bleeding from the soft tissues and bone marrow.

In previous studies, no statistically significant differences were found in the incidence of postoperative infection or thrombosis with using FGHM [20]. In this study, only one patient in the 8 mL group developed pulmonary embolism (PE); however, no statistically significant difference was found in the incidence.

The disadvantage of using FGHM is that it is an expensive biological agent. The results of this study did not show a significant decreased blood loss or clinical outcomes, which suggests that it is not cost-effective.

This study had some limitations. First, this was a prospective historical study and not a randomized controlled trial. Second, the operation time was shorter in the 8 mL group, which may influence the blood loss results. Third, the FGHM dose was comparatively lower (5 mL and 8 mL) than the 10 mL dose used in other studies. Fourth, since all patients received TXA, the unique hemostatic potential of FGHM alone could not be assessed.

## Conclusions

The intraoperative administration of FGHM during TKA was observed to reduce postoperative blood loss when compared to the control group, however, the difference was not statistically significant. Importantly, the use of FGHM did not negatively impact postoperative knee flexion angles nor did it increase the risk of complications, indicating that it is a safe intervention. However, the clinical utility of FGHM as a hemostatic agent for effectively reducing blood loss following primary TKA remains uncertain. Further research is necessary to determine its potential benefits and to establish whether it can offer a significant clinical advantage in blood management during and after TKA procedures.
